# Supplementary figures and images for: Evaluation of the diagnostic performance of laboratory-based c-reactive protein as a triage test for active pulmonary tuberculosis
Source: PLoS One. 2021 Jul 12;16(7):e0254002. doi: 10.1371/journal.pone.0254002 (PMC8274836; doi:10.1371/journal.pone.0254002)

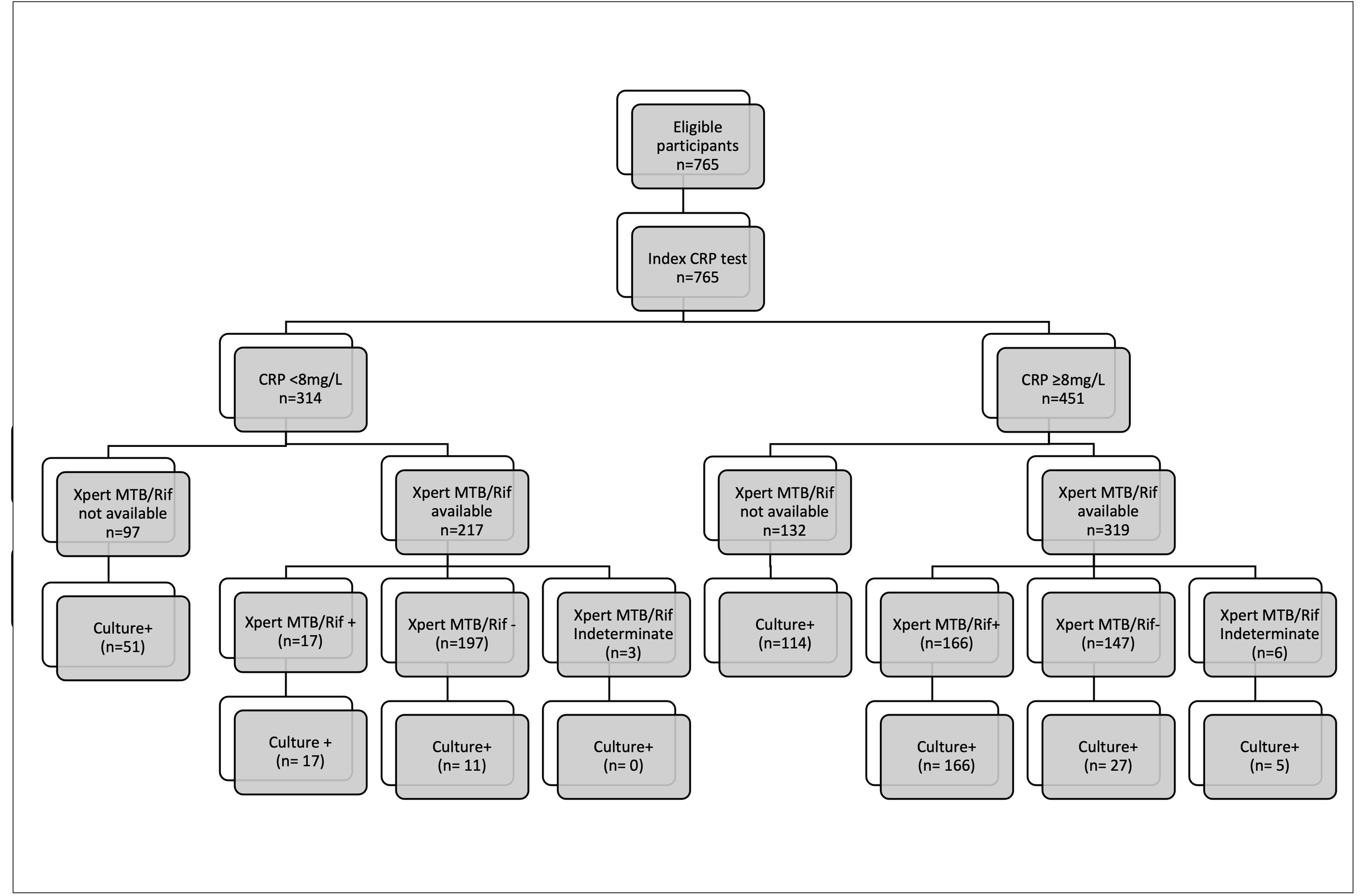

Supplement: S1 Fig — (TIF) [file pone.0254002.s001.tif]

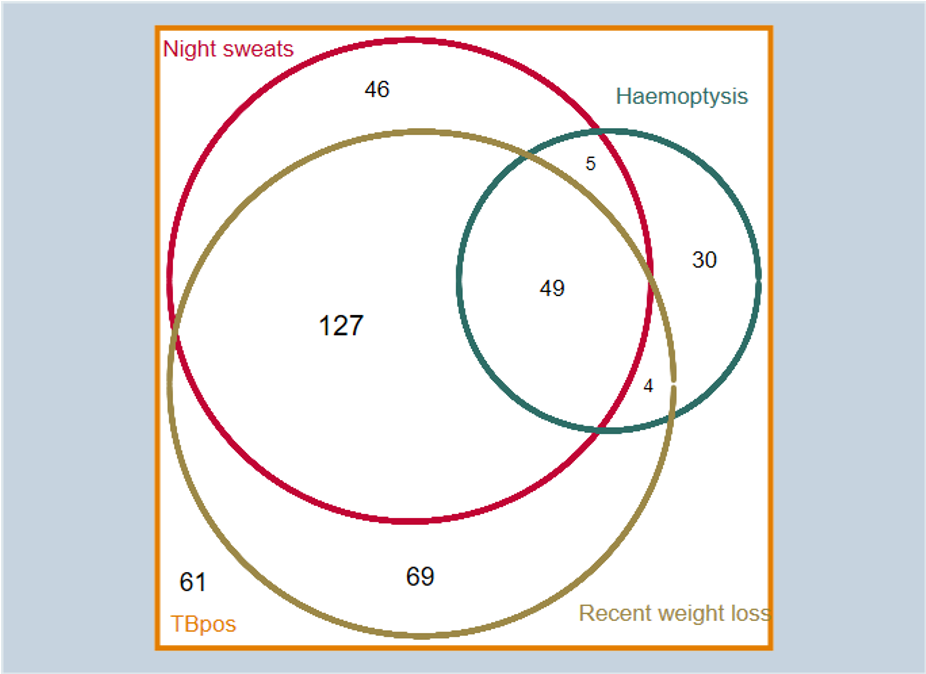

Supplement: S2 Fig — Numbers represent number of participants presenting with the indicated symptom complex. Total TBpos participants presenting with night sweats n = 227, with haemoptysis n = 88, with recent weight loss n = 269 and with none of the above n = 61. (TIF) [file pone.0254002.s002.tif]

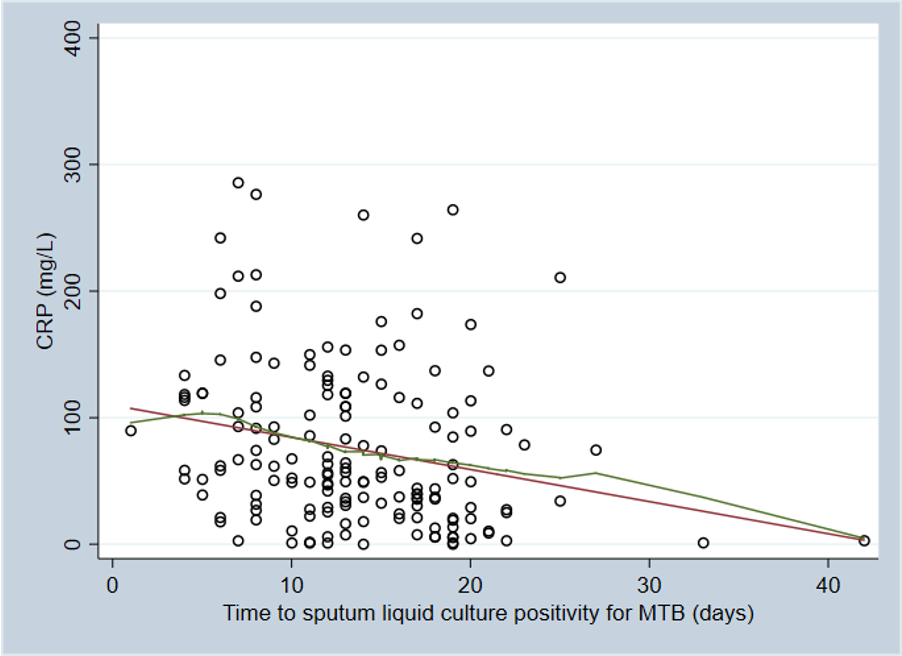

Supplement: S3 Fig — Blue dots represent individual CRP data points, red line demonstrates line of best fit and green demonstrates LOWESS locally weighted smoothing. As CRP concentration decreased linearly with increased time to sputum culture positivity, Pearson’s correlation was applied and found to be significant with a coefficient of -0.234 (p = 0.003). (TIF) [file pone.0254002.s003.tif]

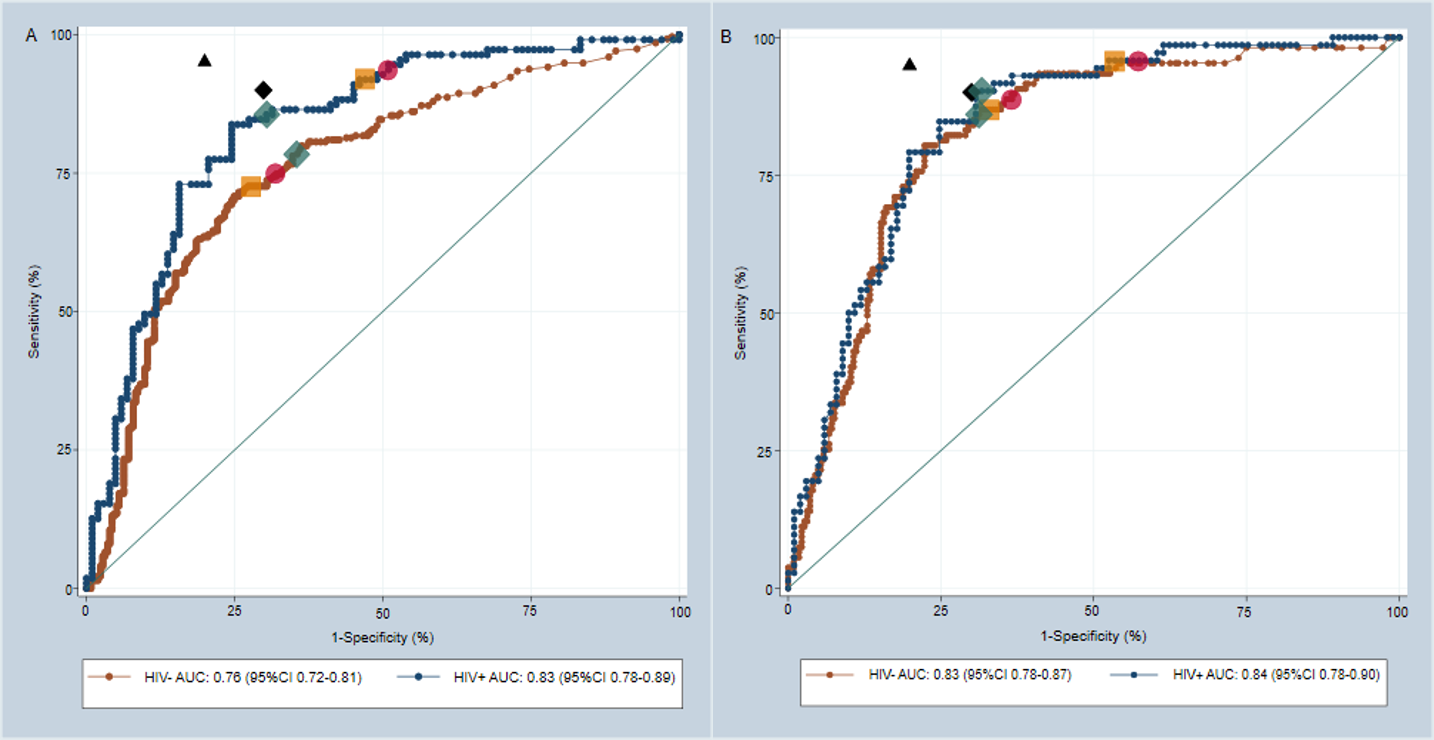

Supplement: S4 Fig — ROC curve analysis of CRP performance in the diagnosis of tuberculosis using A) the MRS and B) the XRS, by HIV status. The shaded orange area represents the sensitivity and specificity combinations that meet at least the minimum target of one of the Target Product Profile characteristics. Minimum (Sens 90% Spec 70%) and Optimal (Sens 95% Spec 80%) Target Product Profile targets are plotted as a black diamond and black triangle respectively. Pre-defined CRP cutoff-points are plotted as red circles and yellow squares with optimal CRP cutoff-points plotted as green diamonds. In A) optimal CRP cutoff-points occurred at 23mg/L for HIV+ participants and 6mg/L for HIV- participants. In B), these cutoff-points were 29mg/L (Sensitivity 90.3% Specificity 68.3%) and 11mg/L (Sensitivity 86.0% and Specificity 68.8%) respectively. AUC = Area Under Curve; ROC = Receiver Operating Characteristic; CRP = C-reactive protein; MRS = Microbiological reference standard; XRS+ Xpert MTB/Rif reference standard. (TIF) [file pone.0254002.s004.tif]

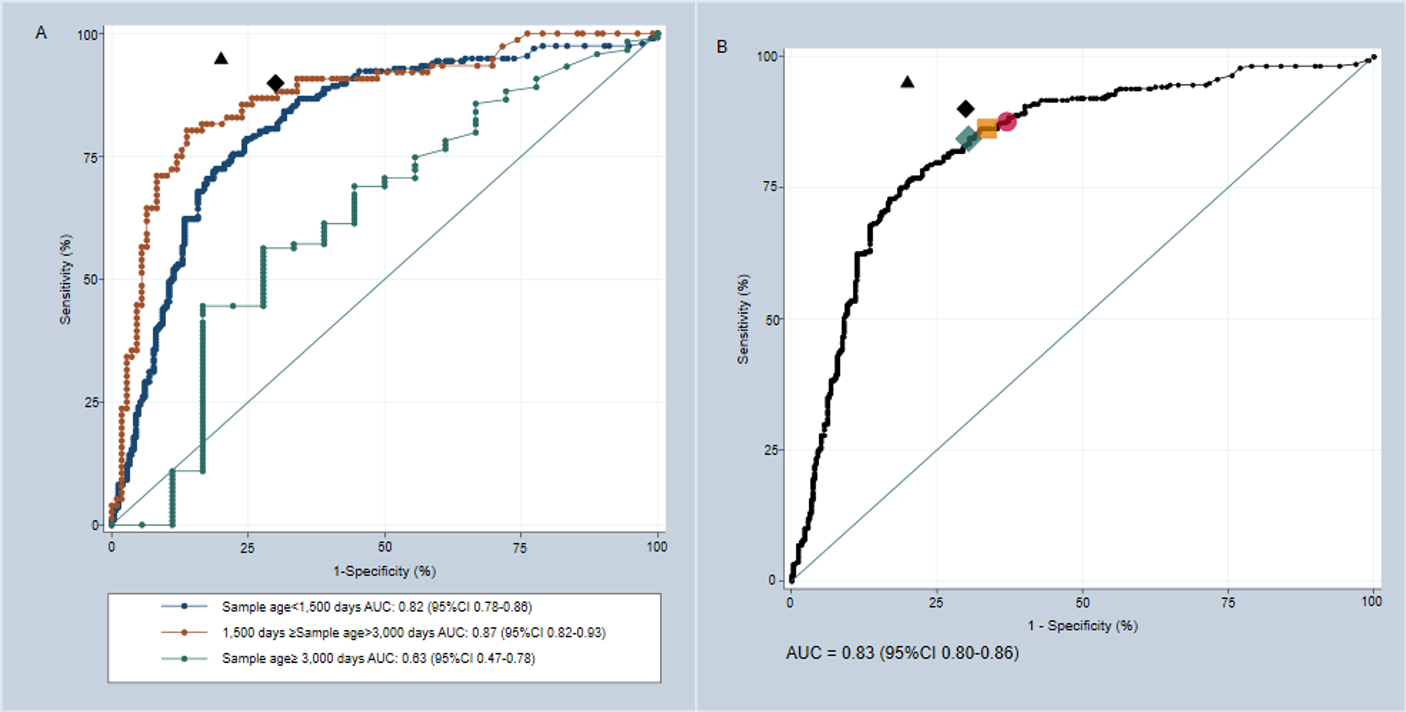

Supplement: S5 Fig — ROC curve analysis of CRP performance in the diagnosis of tuberculosis against the MRS by A) Age of serum sample at time of CRP analysis and B) without Vietnamese sera older than 3,000 days. Variation by study site prompted post-hoc exploratory analysis, which revealed CRP diagnostic performance was significantly worse in pre-2011 samples taken in Viet Nam compared to those taken later. A significant negative correlation between CRP levels and serum sample age was seen in Vietnamese TBpos participants, without a similar correlation being observed in TBneg samples. Optimal sensitivity and specificity against MRS excluding this pre-2011 data (B) was 84.5% (95%CI 79.7–88.3) and 69.5% (95%CI 64.5–74.0) respectively at 12mg/L cutoff-point. This also explains much of the reported difference in CRP performance between the XRS and the MRS, as the pre-2011 Vietnamese participants did not receive Xpert testing. The shaded orange area represents the sensitivity and specificity combinations that meet at least the minimum target of one of the Target Product Profile characteristics. Minimum (Sens 90% Spec 70%) and Optimal (Sens 95% Spec 80%) Target Product Profile targets are plotted as a black diamond and a black triangle respectively. Pre-defined CRP cutoff-points are plotted as red circles (8mg/L) and yellow squares (10mg/L) with optimal CRP cutoff-points plotted as green diamonds. AUC = Area Under Curve; ROC = Receiver Operating Characteristic; CRP = C-reactive protein; MRS = Microbiological Reference Standard. (TIF) [file pone.0254002.s005.tif]
