# Supplementary material for: Evaluation of the diagnostic performance of laboratory-based c-reactive protein as a triage test for active pulmonary tuberculosis
Source: PLoS One. 2021 Jul 12;16(7):e0254002. doi: 10.1371/journal.pone.0254002 (PMC8274836; doi:10.1371/journal.pone.0254002)
Supplement: S1 Table — (PDF) [file pone.0254002.s006.pdf]

Patient Characteristics		N (participants with Xpert MTB/Rif results =527)	
		TB+ (n=183)	TB- (n=344)
**Age**	Median (IQR)	33 (25-42)	39 (31-54)
**Sex**	Female (%)	65 (35.5)	170 (49.4)
	Male (%)	118 (64.5)	174 (50.6)
**Smear status**	S+C+ (% of XRS-defined TB cases)	156 (85.2)	
	S-C+ (% of XRS-defined TB cases)	27 (14.8)	
**HIV status**	Positive (% of patients with known HIV status)	72 (40.2)	101 (31.3)
	Negative (% of patients with known HIV status)	107 (59.8)	224 (68.7)
	Unknown (% of total)	4 (2.2)	19 (5.5)
**CD4 Count**	<200 (% with CD4 count)	24 (61.5)	14 (35.0)
	>=200 (% with CD4 count)	15 (38.5)	26 (65.0)
	Unknown	33	61
	Median CD4 count (IQR)	114 (70-360)	279 (131-475)
**History of BCG**	Positive history of vaccination or scar present (%)	87 (47.5)	182 (52.9)
	Negative history of vaccination (%)	32 (17.5)	76 (22.1)

	Unknown history of vaccination and scar indeterminate (%)	39 (21.3)	64 (18.6)
	Not obtained (%)	25 (13.7)	22 (6.4)
**Prior history of TB**	Positive (%)	48 (26.2)	98 (28.5)
	Negative (%)	132 (72.1)	244 (70.9)
	Unknown (%)	3 (1.6)	2 (0.6)
**QuantiFERON result**	Positive (% with result)	62 (79.5)	5 (4.0)
	Negative (% with result)	12 (15.4)	99 (78.6)
	Indeterminate (% with result)	4 (5.1)	22 (17.5)
	Not obtained	105	218
**Site of Study**	Cambodia (%)	24 (13.1)	24 (7.0)
	Georgia (%)	25 (13.7)	22 (6.4)
	Peru (%)	63 (34.4)	119 (34.6)
	South Africa (%)	54 (29.5)	75 (21.8)
	Viet Nam (%)	17 (9.3)	104 (30.2)
**Number of symptoms at presentation**	1 (%)	7 (3)	101 (33)
	2-3 (%)	109 (49)	142 (46)
	4+ (%)	105 (48)	63 (21)
**Symptom at presentation**	Cough (%)	218 (99)	295 (96)
	Haemoptysis (%)	47 (21)	31 (10)
	Fever (%)	150 (68)	139 (45)
	Night Sweats (%)	156 (71)	120 (39)
	Recent weight loss (%)	165 (75)	134 (44)
