# Supplementary material for: Evaluation of the diagnostic performance of laboratory-based c-reactive protein as a triage test for active pulmonary tuberculosis
Source: PLoS One. 2021 Jul 12;16(7):e0254002. doi: 10.1371/journal.pone.0254002 (PMC8274836; doi:10.1371/journal.pone.0254002)
Supplement: S2 Table — N = total number of cases and controls, n = number of TBpos cases as defined by the XRS. Smear-positive (n = 289) and smear-negative (n = 102) participants were each assessed against all TBneg (n = 374) participants, giving a total of 663 and 476 included participants respectively. (PDF) [file pone.0254002.s007.pdf]

Variable (n/N)		CRP ≥8mg/L		CRP ≥10mg/L		AUC (95%CI)
		Sensitivity (95%CI)	Specificity (95%CI)	Sensitivity (95%CI)	Specificity (95%CI)	
**Overall** *(183/527)*		90.7 (85.6-94.1)	57.3 (52.0-62.4)	89.6 (84.4-93.3)	60.8 (55.5-65.8)	0.83 (0.79-0.87)
**By study site**						
(24/48)	Cambodia	75.0 (55.1-88.0)	29.2 (14.9-49.2)	75.0 (55.1-88.0)	29.2 (14.9-49.2)	0.53 (0.36-0.70)
(25/47)	Georgia	88.0 (70.0-95.8)	63.6 (43.0-80.3)	88.0 (70.0-95.8)	63.6 (43.0-80.3)	0.83 (0.71-0.95)
(63/182)	Peru	90.5 (80.7-95.6)	72.3 (63.6-79.5)	88.9 (78.8-94.5)	76.5 (68.1-83.2)	0.87 (0.81-0.93)
(54/129)	South Africa	96.3 (87.4-99.0)	40.0 (29.7-51.3)	94.4 (84.9-98.1)	44.0 (33.3-55.3)	0.83 (0.76-0.90)
(17/121)	Vietnam	100 (81.6-100)	57.7 (48.1-66.7)	100 (81.6-100)	61.5 (51.9-70.3)	0.90 (0.84-0.96)
**Smear Status**						
(156/500)	Smear + (Xpert+)	92.9 (87.8-96.0)	60.3 (54.8-65.6)	92.3 (87.0-95.5)	63.9 (58.4-69.0)	0.86 (0.83-0.90)
(27/371)	Smear - (Xpert +)	77.8 (59.2-89.4)	57.6 (52.3-62.8)	74.1 (55.3-86.8)	61.2 (55.9-66.2)	0.71 (0.64-0.79)
**HIV status**						
(72/173)	Positive	95.8 (88.5-98.6)	42.6 (33.3-52.3)	95.8 (88.5-98.6)	46.5 (37.1-56.2)	0.84 (0.78-0.90)
(107/331)	Negative	88.8 (81.4-93.5)	63.4 (56.9-69.4)	86.9 (79.2-92.0)	67.0 (60.0-72.8)	0.83 (0.78-0.87)

Number of presenting symptoms						
(7/108)	1 symptom	66.7 (20.8-93.9)	77.1 (68.2-84.1)	66.7 (20.8-93.9)	80.0 (71.4-86.5)	0.71 (0.38-1.00)
(109/251)	2-3 symptom	87.4 (78.8-92.8)	57.3 (49.7-64.6)	85.1 (76.1-91.1)	60.4 (52.7-67.5)	0.79 (0.73-0.85)
(105/168)	4+ symptom	94.6 (88.0-97.7)	29.3 (20.2-40.4)	94.6 (88.0-97.7)	34.7 (24.9-45.9)	0.79 (0.72-0.85)
