# Supplementary material for: Evaluation of the diagnostic performance of laboratory-based c-reactive protein as a triage test for active pulmonary tuberculosis
Source: PLoS One. 2021 Jul 12;16(7):e0254002. doi: 10.1371/journal.pone.0254002 (PMC8274836; doi:10.1371/journal.pone.0254002)
Supplement: S3 Table — (PDF) [file pone.0254002.s008.pdf]

Variable		CRP cutoff-point (mg/L)							
		**2**	**4**	**6**	**8**	**10**	**12**	**14**	**18**
**Overall**	Sensitivity	96.2	95.1	93.4	90.7	89.6	86.9	85.3	84.7
	Specificity	30.2	42.4	52.9	57.3	60.8	64.0	65.7	69.5
		**2**	**4**	**6**	**8**	**10**	**12**	**20**	**30**
**HIV+**	Sensitivity	98.6	98.6	98.6	95.8	95.8	94.4	93.1	87.5
	Specificity	16.8	26.7	38.6	42.6	46.5	48.5	60.4	69.3
**HIV-**	Sensitivity	95.3	93.5	91.6	88.8	86.9	84.1	80.4	71.0
	Specificity	36.6	50.9	59.8	63.4	67.0	70.6	77.7	82.6
