# Supplementary material for: Evaluation of the diagnostic performance of laboratory-based c-reactive protein as a triage test for active pulmonary tuberculosis
Source: PLoS One. 2021 Jul 12;16(7):e0254002. doi: 10.1371/journal.pone.0254002 (PMC8274836; doi:10.1371/journal.pone.0254002)
Supplement: S4 Table. Sensitivity and specificity of CRP against the MRS by study site. N = total number of cases and controls, n = number of TBpos cases as defined by the MRS — (PDF) [file pone.0254002.s009.pdf]

**Study Site** *(n/N)*		**CRP ≥8mg/L**		**CRP ≥10mg/L**		**AUC (95%CI)**
		**Sensitivity (95%CI)**	**Specificity (95%CI)**	**Sensitivity (95%CI)**	**Specificity (95%CI)**	
(30/48)	Cambodia	76.7 (59.1-88.2)	33.3 (16.3-56.3)	76.7 (59.1-88.2)	33.3 (16.3-56.3)	0.59 (0.42-0.77)
(30/47)	Georgia	80.0 (62.7-90.5)	64.7 (41.3-82.7)	80.0 (62.7-90.5)	64.7 (41.3-82.7)	0.74 (0.59-0.86)
(125/263)	Peru	87.2 (80.2-92.0)	79.7 (72.2-85.6)	86.4 (79.3-91.3)	83.3 (76.2-88.6)	0.90 (0.86-0.94)
(70/140)	South Africa	95.7 (88.1-98.5)	44.3 (33.2-55.9)	91.4 (82.5-96.0)	47.1 (35.9-58.7)	0.81 (0.74-0.89)
(136/2670	Vietnam	65.4 (57.1-72.9)	58.8 (50.2-66.8)	62.5 (54.1-70.2)	62.6 (54.1-70.4)	0.64 (.58-0.71)
