# Supplementary material for: Evaluation of the diagnostic performance of laboratory-based c-reactive protein as a triage test for active pulmonary tuberculosis
Source: PLoS One. 2021 Jul 12;16(7):e0254002. doi: 10.1371/journal.pone.0254002 (PMC8274836; doi:10.1371/journal.pone.0254002)
Supplement: S5 Table — (PDF) [file pone.0254002.s010.pdf]

Variable		CRP cutoff-point (mg/L)							
		**2**	**4**	**6**	**8**	**10**	**12**	**14**	**18**
**Overall**	Sensitivity (95%CI)	90.3	85.4	83.1	79.8	77.8	76.0	73.4	70.3
	Specificity (95%CI)	35.6	48.1	58.8	62.8	66.0	69.5	70.6	73.8
		**2**	**4**	**6**	**8**	**10**	**12**	**18**	**24**
**HIV+**	Sensitivity (95%CI)	97.3	96.4	96.4	93.7	91.9	91.0	86.5	84.7
	Specificity (95%CI)	21.6	33.3	46.1	49.0	52.9	54.9	59.8	69.6
**HIV-**	Sensitivity (95%CI)	88.0	81.4	78.5	74.8	72.6	70.8	64.6	58.8
	Specificity (95%CI)	41.5	55.3	64.4	68.0	71.2	75.1	78.7	83.0
