# Supplementary material for: Evaluation of the diagnostic performance of laboratory-based c-reactive protein as a triage test for active pulmonary tuberculosis
Source: PLoS One. 2021 Jul 12;16(7):e0254002. doi: 10.1371/journal.pone.0254002 (PMC8274836; doi:10.1371/journal.pone.0254002)
Supplement: S6 Table — In this algorithm, a positive CRP triage test above the stated threshold would trigger patients to move forward to confirmatory testing by the stated method. The performance of chest X-ray (CXR) as a triage test was assessed for comparison. A CXR was defined as positive for TB if the radiographic appearance was judged to be consistent with typical or atypical TB. The sensitivity and specificity of a single Xpert MTB/Rif against the MRS is also presented. (PDF) [file pone.0254002.s011.pdf]

Triage algorithm		Observations (n)	% of n requiring a confirmatory test	Sensitivity (95%CI)	Specificity (95%CI)
**Xpert MTB/Rif alone**		527	100%	82.8 (77.3-87.2)	100 (98.8-100)
**Xpert Confirmation**					
CRP threshold (mg/L)	10	527	56.7%	74.2 (68.1-79.5)	100 (98.8-100)
	8	527	59.4%	75.1 (69.0-80.4)	100 (98.8-100)
	6	527	63.2%	77.4 (71.4-82.4)	100 (98.8-100)
	4	527	70.6%	78.7 (72.9-83.6)	100 (98.8-100)
	2	527	78.9%	79.6 (73.8-84.4)	100 (98.8-100)
**Liquid Culture confirmation**					
CRP threshold (mg/L)	10	765	56.3%	77.7 (73.4-81.6)	100 (99.0-100)
	8	765	59.0%	79.8 (75.5-83.5)	100 (99.0-100)
	6	765	62.6%	83.1 (79.1-86.5)	100 (99.0-100)
	4	765	69.0%	85.4 (81.6-88.6)	100 (99.0-100)
	2	765	77.6%	90.3 (86.9-92.8)	100 (99.0-100)
**Chest X-ray triage**					
CXR alone		673	N/A	93.2(90.1-95.5)	63.1(57.8-68.1)
CXR Xpert MTB/Rif		463	57.9%	76.8(70.3-82.3)	100 (98.6-100)
CXR Liquid Culture		673	65.4%	93.2(90.1-95.5)	100 (98.9-100)
