# Supplementary material for: Evaluation of the diagnostic performance of laboratory-based c-reactive protein as a triage test for active pulmonary tuberculosis
Source: PLoS One. 2021 Jul 12;16(7):e0254002. doi: 10.1371/journal.pone.0254002 (PMC8274836; doi:10.1371/journal.pone.0254002)
Supplement: S7 Table — All variables with a p value <0.1 were considered for inclusion into multivariable analysis. Akaike Information Criterion values were then used to build the best model. CD4 count was excluded from the multivariable model despite meeting significance as only 95 study participants had recorded values. AIC was not reported for CD4 count as sample size differed from other variables. (PDF) [file pone.0254002.s012.pdf]

Variable		Fold-change in CRP (unadjusted)	95% Conf. Interval		p-value	AIC
			Lower	Upper		
**TB status**	TB+	5.81	4.57	7.36	<0.001	2907
	TB-	1.0				
**Smear microscopy result**	Smear+	6.36	5.00	8.08	<0.001	2898
	Smear-	1.0				
**HIV status**	HIV+	2.48	1.85	3.32	<0.001	2969
	HIV-	1.0				
**Sex**	Female	0.51	0.39	0.67	<0.001	3091
	Male	1.0				
**Number of symptoms at presentation**	4+	10.07	7.17	14.15	<0.001	2939
	2-3	3.10	2.27	4.22	<0.001	
	1	1.0				
**Study site**	Cambodia	2.48	1.40	4.39	0.002	3067
	South Africa	2.66	1.84	3.90	<0.001	
	Georgia	1.52	0.86	2.70	0.146	
	Vietnam	0.99	0.72	1.35	0.93	
	Peru	1.0				
**Age**	/year increase	0.99	0.98	1.00	0.02	3091
**CD4 count (n=95)**	≥200	0.38	0.19	0.76	0.007	-
	<200	1.0				
