# Supplementary material for: Evaluation of the diagnostic performance of laboratory-based c-reactive protein as a triage test for active pulmonary tuberculosis
Source: PLoS One. 2021 Jul 12;16(7):e0254002. doi: 10.1371/journal.pone.0254002 (PMC8274836; doi:10.1371/journal.pone.0254002)
Supplement: S1 File — (DOCX) [file pone.0254002.s013.docx]

**Study Protocol**

**TB Triage Test – CRP Evaluation**

**Background**

Working in the direction of developing an easy-to-use point-of-care test to rule-out tuberculosis (TB), FIND has identified several host biomarker signatures that showed promising performance with respect to the target product profile (TPP). However, CRP gained some traction as an individual marker for ruling-out TB. Work of C. Yoon showed that CRP can be used as an effective TB screening test among people living with HIV (PLHIV). Using a cut-point of 8 mg/L, POC CRP had 90% sensitivity and 70% specificity in reference to two liquid cultures among PLHIV with CD4 ≤350 cells/μL who presented at HIV clinics in Uganda (Yoon, Semitala, et al., 2017b). In a meta analysis, C. Yoon also reported that, among out-patients, CRP had high sensitivity (93%, 95%CI 88–98) and moderate specificity (60%, 95%CI 40–75) for active pulmonary TB. Specificity was lowest among in-patients (21%, 95%CI 6–52) and highest among out-patients undergoing TB screening (range 58–81%). There was no difference in summary estimates by HIV status (Yoon, Chaisson, et al., 2017a). Recently, C. Yoon reported that, for HIV-infected adults with CD4 counts ≤350 cells/μL, replacing symptom-based screening (current recommendation) with point-of-care CRP-based TB screening could improve the efficiency and reduce the cost of ICF, without compromising diagnostic yield (Yoon et al., 2018).

The TPP for a TB triage test is very challenging to meet due to the competitive requirements of low cost and high performances (sensitivity of 90% and specificity of 70%). A single biomarker signature would allow a reduction in technical complexity and therefore would lower the overall cost. CRP is therefore an appealing candidate. CRP has the advantage of being measurable with commercially available “IVD-grade” point-of-care tests from capillary whole blood at a cost of about USD 2.

The CRP evaluation in the work of C. Yoon represented a specific population (PLHIV) from a unique country (Uganda). On the other hand, the meta-analysis encompassed 1793 adults, 72% of whom had confirmed HIV infection from 5 countries (South Africa N=5, Uganda N=1, India N=1, South Korea N=2, United Kingdom N=1). Because patients self-presenting with TB symptoms have a higher prevalence of pyogenic infections or other systemic inflammatory conditions mimicking TB, the specificity of CRP for active PTB can be expected to be lower in this population than in populations undergoing provider-initiated TB screening, such as PLHIV presenting for routine HIV/AIDS care.

Therefore, FIND would like now to characterize further the concentration level of this host marker candidate in serum samples from FIND collection using samples representing the global TB epidemics and within individuals that self-present with TB symptoms.

**Objectives**

To measure the concentration level of CRP in serum samples from FIND bio-bank with the purpose to assess CRP diagnostic performances in the context of a TB rule-out test (i.e. in individuals that self-present with TB symptoms, passive screening). A second objective is to understand whether a unique cut-off can be used across populations.

**Sample collection**

For this retrospective case-control study, a total of 765 serum samples were selected from FIND’s biobank. These samples were previously collected in studies from adults presenting at primary care sites in Cambodia, Georgia, Peru, South Africa and Vietnam with clinical symptoms of TB, but not receiving TB treatment at the time of sample collection. Approval by local Ethics Committee and informed patient consent was obtained before enrolling patients and no personally identifiable information was available to FIND or to the researchers.

**Classification of patients and composite reference standard**

Patient were classified using a composite reference standard on the basis of clinical and laboratory findings as described elsewhere (Broger et al. 2017). TB-positive (TB+) were patients with at least one positive culture. Participants who were smear negative and culture negative on ≥4 cultures on all sputum samples and who exhibited symptoms resolution in the absence of tuberculosis treatment and negative sputum culture results at follow-up visit were classified as TB-. Subjects were further classified as HIV+ or HIV- based on HIV rapid tests.

**Sample size and selection estimates**

Inclusion criteria included patients classified as TB-positive and TB-negative, therefore excluding all other cases (e.g. clinical TB, likely subclinical TB, possible TB, non-TB without FU).

Per site, we would need 65 TBpos and 140 TBneg are required to achieve a total width of the 95% confidence interval of 15% based on a 90% target for sensitivity (95%CI: 83 to 97) and a 70% target for specificity (95%CI: 63 to 77). Therefore, we have measured:

**Host marker quantitation**

CRP concentration has been measured by an IVD-grade assay on Abbott Architect C8000 at Quest laboratories.

**Ethical Approval**

All study-related activities were approved by the Human Research Ethics Committees (HREC) of the partners in-countries. These were: University of Cape Town, South Africa; Universidad Peruana Cayetano Heredia, Peru; Pham Ngoc Thach Hospital, Vietnam; Calmette Hospital, Cambodia; National Centre for Tuberculosis and Lung Diseases, Georgia.

**Analysis Plan**

The following are expected to be produced:

- A description of participant characteristics.
- Distribution of CRP concentration:
  - Dot plots of CRP concentration (TB+ vs TB-; S-C+ vs S+C+;  by number of symptoms; within those groups across sites)
- Multivariable analysis describing the relation of variables to CRP concentration
- Sensitivity and Specificity analysis:
  - Against the microbiological reference standard
    - ROC curve (pooled, by HIV status)
      - Including threshold analysis to determine optimal cutoff-points by manual data inspection
    - Sensitivity/specificity (pooled, by site, by smear-status, by HIV status, by number of symptoms at presentation)
      - At cut-off 8 mg/l
      - At cut-off 10 mg/l
  - Against the Xpert reference standard
    - ROC curve (pooled, by HIV status)
      - Including threshold analysis to determine optimal cutoff-points by manual data inspection
    - Sensitivity/specificity (pooled, by site, by smear status, by HIV status, by number of symptoms at presentation)
      - At cut-off 8 mg/l
      - At cut-off 10 mg/l
- The performance of an algorithm in which a positive CRP triage test is followed by a confirmatory test, either a single Xpert MTB/Rif test or a single liquid MGIT culture test.
- To plot time to liquid culture positivity against CRP result and interrogate for correlation by LOWESS

**Bibliography**

Yoon, C., Chaisson, L. H., Patel, S. M., Allen, I. E., Drain, P. K., Wilson, D., & Cattamanchi, A. (2017a). Diagnostic accuracy of C-reactive protein for active pulmonary tuberculosis: a meta-analysis. *The International Journal of Tuberculosis and Lung Disease*, *21*(9), 1013–1019. http://doi.org/10.5588/ijtld.17.0078

Yoon, C., Semitala, F. C., Asege, L., Katende, J., Mwebe, S., Andama, A. O., et al. (2018). Yield and Efficiency of Novel Intensified Tuberculosis Case-Finding Algorithms for People Living with HIV. *American Journal of Respiratory and Critical Care Medicine*. http://doi.org/10.1164/rccm.201803-0490OC

Yoon, C., Semitala, F. C., Atuhumuza, E., Katende, J., Mwebe, S., Asege, L., et al. (2017b). Point-of-care C-reactive protein-based tuberculosis screening for people living with HIV: a diagnostic accuracy study. *The Lancet Infectious Diseases*, *17*(12), 1285–1292. http://doi.org/10.1016/S1473-3099(17)30488-7
