# Supplementary material for: Evaluation of the diagnostic performance of laboratory-based c-reactive protein as a triage test for active pulmonary tuberculosis
Source: PLoS One. 2021 Jul 12;16(7):e0254002. doi: 10.1371/journal.pone.0254002 (PMC8274836; doi:10.1371/journal.pone.0254002)
Supplement: Attachment — Submitted filename: CRP for TB Triage Reviewer comments with replies v1.2.docx [file pone.0254002.s015.docx]

Foundation for Innovative Novel Diagnostics Headquarters

Campus Biotech

9 Chemin des Mines

1202 Geneva

Switzerland

08.06.20

Dear Editor of PLOS One,

It is with pleasure that we are submitting our revised article to PLOS One entitled:

**Evaluation of the diagnostic performance of laboratory-based C-reactive protein as a triage test for active pulmonary tuberculosis**

Manuscript ID number: PONE-D-20-19334R1

We would like to thank the reviewers for their valuable time and useful contributions to the manuscript. We have carefully considered and addressed each of the comments provided and have made revisions in line with their recommendations. Herein, we are presenting a more comprehensive and improved manuscript for publication.

Point-by-point responses to each of the reviewers’ comments are included within the revised submission. We thank you for considering our revised manuscript for publication and look forward to your reply.

Yours sincerely,

Samuel Schumacher

Deputy Head of TB

FIND

[samuel.schumacher@finddx.org](mailto:samuel.schumacher@finddx.org)

T +41 (0) 22 710 05 91

M +41 (0) 79 911 45 91

Major points:

| Point | Reviewer | Line | Reviewer Comment | Response |
| --- | --- | --- | --- | --- |
| 1 | One | 110-119 | How were controls selected? Were controls unmatched? From line 159 it seems they were matched on HIV status. | This point has been addressed by further clarifying how cases and controls were selected and the fact that they were matched by HIV status in the relevant section of the methods. |
| 2 | One | 175-177 | I don't think it's appropriate to conduct a linear regression on case-control data without correcting for selection bias associated with oversampling of cases. For example, see doi: 10.1093/biostatistics/kxt041 | We believe that this methodology proposed by the reviewer is designed to address circumstances in which cases and controls are selected with unequal probability and therefore we are not sure that it applies to our study. |
| 3 | One | N/A | Additionally, with a data-driven variable selection procedure for your model, please also include stability / sensitivity analyses. See doi:10.1002/bimj.201700067 | We agree with the reviewer that in data-driven variable selection a sensitivity analysis is an important part of best-practice methodology. However, as the number of variables we included a priori in the univariate part of our analysis was small (8 in total), we felt that carrying out a full sensitivity analysis to justify our variable selection wasn't necessary. |
| 4 | One | 171-172 | Similarly, the ROC analysis should be adjusted for the case-control design. See doi: 10.1016/j.acra.2013.03.004, and doi: 10.1373/clinchem.2012.186007 | As with the point above on our linear regression, we believe that this methodology proposed by the reviewer is designed to address circumstances in which cases and controls are selected with unequal probability and therefore we are not sure that it applies to our study. |
| 5 | Two | N/A | Your paper makes it clear that you are concerned with CRP in conjunction with another test, which you expect will usually be Xpert MTB/Rif (but could be culture). So in referring to the TPP specified minimum sensitivity and specificity, should you not be looking at the figures for the composite of CRP followed (if threshold is met) by Xpert, and not CRP alone? If one can choose the appropriate CRP threshold to obtain high enough sensitivity, and then follow it with Xpert, why should we demand high specificity of CRP alone? Your table S6 notes that with Xpert as the second part of this composite CRP-Xpert testing one sees 100% specificity. You report that CRP fails to meet the TPP specified requirement of at least 70% specificity but in this paper you are not suggesting CRP alone. Even a CRP specificity less than 70% could result in some reduction of Xpert testing (the only benefit of high CRP specificity in your composite testing model seems to be a health system benefit - as opposed to improved diagnostic yield which is not diminished by the low specificity of CRP, when used with Xpert or culture). | We agree with the reviewers point that the overall performance of a two-step algorithm is highly important to consider. However, our work is designed to focus specifically on the question of performance related to first step of that algorithm and, as the reviewer states in the second paragraph, its health systems benefit by rationalising the use of confirmatory testing. The WHO target product profile for a triage test is designed to be in reference to the confirmatory test chosen and so is a good standard for our research question. The 70% specificity target for a triage test in this instance accounts for the fact that at lower prevalence settings a lesser specificity would cause the number of false positives to exceed the number of true positives. This would erode the health system benefit to the point that they may be outweighed by the potential loss of trust in the triage test. We have altered the manuscript to try and make this aim clearer. |
| 6 | Two | N/A | In your discussion of the sensitivity of CRP among HIV+ and HIV- persons with TB you note the use of different threshold values for HIV+ persons, or the possible addition of clinical features to an algorithm. No mention is made of the possibility of more than one CRP measurement per patient during the diagnostic evaluation period. Your analysis and others make it clear that CRP is more likely to be elevated when there are clinical (eg., fever) and pathological (eg., hemoptysis, indicative of cavitation) features of intense inflammation, or its sequelae. It is likely that symptoms become more obvious the longer one has untreated TB, but sequential changes in CRP among persons suspected of having TB have not, as far as I am aware, been examined. Work by Stephen Lawn and others has looked at sequential CRP in response to TB treatment which may offer a diagnostic confirmation when a fall in CRP is observed. | We agree with the reviewer on this point that this represents a very interesting line of enquiry and one that should be pursued. Unfortunately, we do not have the data to be able to pursue it in this work. However, we have altered the discussion to make mention of it. |
| 7 | Three | N/A | However, my main concern with this conclusion is that TB triage testing would not be applied to individuals with any TB symptom, unless they were at high risk for TB (e.g., HIV-positive). For everyone else, TB triage testing would be applied to patients with cough ≥2 weeks, however CRP diagnostic accuracy data for this group was not presented in this analysis and is likely to yield different diagnostic accuracy results. If symptom duration data is available, diagnostic accuracy of CRP should be assessed in the appropriate target populations for triage testing (cough ≥2 weeks if HIV-neg, any TB symptom in the past 1 month if HIV-pos). | We agree with the reviewer that triage testing would usually be offered to the patient population they specify. However, in this work we tried to cast a wider net for our primary analysis and chose current cough as opposed to cough ≥2 weeks as this was shown to be a more sensitive screening algorithm for PTB in the following paper: https://www.ncbi.nlm.nih.gov/pmc/articles/PMC3391193/  A sub-analysis to assess the diagnostic performance of CRP in the target populations specified by the reviewer would be a good addition to our manuscript but unfortunately, we do not have sufficient granularity in our symptom data to do so. |
| 8 | Three | N/A | TB test nomenclature - Because the TB literature has used 'triage test' to refer to both primary screening tests (a test applied to an unselected group to identify individuals with suspected disease requiring confirmatory testing) as well as secondary screening tests which is the conventional definition (a test applied to individuals with suspected to disease with the goal of reducing the proportion requiring confirmatory testing), it would be helpful for the authors to be very explicit with how this manuscript defines triage test. -- Abstract - please consider clarifying definition of triage test here. Currently, the first sentence could also be used to describe a primary screening test. - Intro, 2nd paragraph - I'm not familiar with the term 'two-stage diagnostic algorithm,' used here and find it a bit confusing. Seems like 'two-stage diagnostic algorithm' doesn't include cough ≥2 weeks (screening test) but describes triage testing and confirmatory testing when applied to patients with cough ≥ 2 weeks. Please consider using conventional terminology/nomenclature and clearly defining these when more than 1 definition is commonly used. | We agree with the reviewer that our use of ‘triage test’ and ‘two-stage diagnostic algorithm’ in the manuscript is not specific enough as it stands and we have altered the relevant sections in the abstract and main text to make them so, in line with their suggestion. |
| 9 | Three | N/A | Geographic differences in CRP diagnostic accuracy - CRP sensitivity was lower in Asian countries (Cambodia, Vietnam). It would be helpful to discuss this a bit more in the discussion. Marc Lipman (<https://bmcinfectdis.biomedcentral.com/articles/10.1186/s12879-016-1612-1>) reported similar findings: Asian ethnicity was associated with lower median CRP levels among those with TB. -- Tables S2 and S4 - why is CRP diagnostic accuracy for Vietnam so different in Table S2 and S4? | We agree with the reviewer’s point here and have expanded this paragraph in the discussion to include the suggested reference. Sadly, we do not have strain data for our patients to explore this line of enquiry further.  Regarding the reviewers point about the difference between Vietnamese data in Table S2 and S4 – Xpert MTB/Rif testing was carried out only on a more recent subgroup of the total Vietnamese cohort – this data is presented in S2. Culture data for the total Vietnamese cohort is presented in S4. We did further analysis to interrogate this difference. Culture performance in the subgroup presented in S2 was better than for the cohort as a whole in S4, although the difference was less marked that presented between S2 and S4. We conducted some analysis as to the effect of age of sample in the cohort as a whole and found no significant correlation between age and CRP levels. However, there was a signal when this analysis was applied to the Vietnamese data alone. Against a sample-degradation hypothesis, CRP has been shown to be stable in serum for extended periods of time DOI: 10.1016/j.clinbiochem.2013.12.014. We feel it is possible there is some age-related effect here, but that also the overall performance of CRP in Vietnam is also related to host factors and strain time as laid out in the paper the reviewer mentions. |

Minor points:

| Point | Reviewer | Line | Reviewer Comment | Response |
| --- | --- | --- | --- | --- |
| 1 | One | 29 | no study has assessed its suitability as a triage test." Similarly stated on line 353. What about doi: 10.1097/QAD.0000000000001902, and doi: 10.5588/ijtld.13.0519 ? | We agree with the reviewer that the referenced work has investigated CRP as a triage test in HIV+ participants but that there isn’t a dedicated study we are aware of that looks at HIV-negative patients. We have amended the manuscript to reflect this. |
| 2 | One | 83-89 | Citation | We agree with the reviewer and have actioned this point |
| 3 | One | 165 | Parametric is a general term for any probability distribution. Do you mean normally distributed? How was this determined? | We agree with the reviewer and have changed this to ‘normally-distributed’ in the manuscript |
| 4 | One |  | Table 1: Please include Xpert status for cases | We agree with the reviewer and have actioned this point |
| 5 | One | 361 | 50% generally reported in clinical practice" This needs a citation. Maybe in HIV + cohorts, but generally? | We agree with the reviewer and have cited this point |
| 6 | Two | 37-38 and 104-106 | Line 37-38: The abstract states that "The PTB reference standard was a contemporaneously collected single sputum MGIT culture result." Line 104-106: Later in Materials and Methods it is stated that "To be included in the analysis all participants had to provide both a blood sample and at least 2 sputum samples at enrolment prior to initiation of anti-tuberculous therapy." Is the reference standard for diagnosis of TB one or two MGIT TB cultures? If two, are they performed on different specimens | We agree with the reviewers point that these statements in the manuscript are somewhat contradictory. We have amended the abstract to reflect the methods – the reference standard is two MGIT as is now stated in the Methods. |
| 7 | Two | 311 | However, the suboptimal specificity might make the implementation of the test in an Xpert-algorithms impossible." The word 'algorithm' should be singular (or the word 'an' should be removed if the plural is intended). | We agree with the reviewer and have actioned this point |
| 8 | Three | 78 | Should change "PTB outpatients" to "outpatients with suspected TB (or outpatients with TB symptoms)." | We agree with the reviewer and have actioned this point |
| 9 | Three | 82-83 | Are prior studies limited when the reference cited is a meta-analysis that includes 6 studies that included HIV-negative patients? Also, please consider briefly (in 1 or 2 sentences) summarizing the results of this SR/MA that are relevant to this manuscript, which would provide readers with some context for the following 2 sentences. | We agree with the reviewer and have actioned this point, changing the wording of the relevant part of the manuscript and summarising the results of this SR. |
| 10 | Three | 83-85 | This sentence suggests that CRP specificity will be lower among HIV-negative patients, is that the intent? I would assume the opposite because of lower rates of infection relative to HIV-positive patients. | We agree with the reviewer that this section as it stands didn’t follow. We have amended it accordingly. |
| 11 | Three | 85-86 | Individuals who present to healthcare with symptoms suggestive of PTB (therefore requiring triage instead of screening)..." With the current TB testing algorithm, patients with TB symptoms require confirmatory testing. I understand the point being made is that CRP triage testing can be expected to have reduced specificity relative to CRP screening but I think the comment in parentheses obscures this point | We agree with the reviewer and have actioned this point to make it clearer. |
| 12 | Three | 90 | Should the last reference in paragraph 3 (ref 13) be deleted here? | We agree with the reviewer and have actioned this point |
| 13 | Three | 99-101 | Please provide more detail about the study population (e.g., outpatients, inpatients or both?) | We agree with the reviewer that more detail is required and have amended the manuscript |
| 14 | Three | 101-104 | Same as above major comment: If symptom duration data are available, please pull out cough ≥2 weeks and any 1/4 TB symptom in past month. | As we have mentioned above in response to the major comment, whilst we agree with the reviewer that this would be a worthy analysis, we do not have the data to complete it. |
| 15 | Three | Methods | Last sentence of introduction mentions that a high sensitivity CRP (hsCRP) assay was used. The methods section does not mention this in the Index test section. Prior studies of CRP in TB use a standard sensitivity assay. Can the authors explain why a high sensitivity assay was chosen and whether the type of assay (hs or standard) would impact results | We agree with the reviewers point that there is a discrepancy here. The analyser employed in this study does have the ability to process hsCRP. However, for this study we employed a standard sensitivity mode. The manuscript has been amended to reflect this and resolve the discrepancy. |
| 16 | Three | Table 1 | TB symptoms at presentation. Same as 2nd Methods comment. Also, if presence of at least 1 TB symptom was a requirement for this analysis and no patients had 0 symptoms, shouldn't the number of symptoms at presentation begin with 1 (opposed to '0-1')? | We agree with the reviewers point here – the 0-1 symptoms at presentation were a mistake – this should be 1 symptom. We have amended the manuscript and figures accordingly |
| 17 | Three | Page 17, 2nd paragraph | how were optimal cut-points determine? by AUROC? If so, this approach weighs sensitivity and specificity equally, but clinically, most ppl would feel that 1 missed case of active TB far outweighs a 1 false-positive case. From this clinical perspective, it might make more sense to fix sensitivity at 90% to identify optimal CRP cut-point. FYI: the manuscript also mentions alternative CRP cut-points in the Discussion (line 288-290, 324 paragraph). | We agree with the reviewers point that further clarity is required as to the methodology employed to calculate optimal cutoff-points. We have now amended the methods section of the manuscript to make this clearer. Sensitivity and specificity were weighed equally in the TPP of the WHO and we feel that the importance of one versus the other depends on the TB prevalence in a given setting. As a result, we have gone with the methodology above to specify optimal cutoff-points as it lends weight to the impact of both aspects of test performance. |
| 18 | Three | Page 17, 3rd paragraph | related to major comment above. Recommend using CRP triage testing as it would be applied in currently recommended TB testing algorithms (e.g., HIV negative patients with cough≥ 2 weeks) | We have expanded on the reasoning behind our decision-making in regard to this point in the response to the major comment above. |
| 19 | Three | 298 | replace 'conversion' with 'positivity'? | We agree with the reviewer and have actioned this point |
| 20 | Three | 302-303 | this sentence is unclear | We agree with the reviewer and have actioned this point |
