# Supplementary material for: Evaluation of the diagnostic performance of laboratory-based c-reactive protein as a triage test for active pulmonary tuberculosis
Source: PLoS One. 2021 Jul 12;16(7):e0254002. doi: 10.1371/journal.pone.0254002 (PMC8274836; doi:10.1371/journal.pone.0254002)
Supplement: Attachment — Submitted filename: CRP for TB Triage Reviewer comments with replies v2.0.docx [file pone.0254002.s016.docx]

Foundation for Innovative Novel Diagnostics Headquarters

Campus Biotech

9 Chemin des Mines

1202 Geneva

Switzerland

08.05.21

Dear Editor of PLOS One,

It is with pleasure that we are submitting our revised article to PLOS One entitled:

**Evaluation of the diagnostic performance of laboratory-based C-reactive protein as a triage test for active pulmonary tuberculosis**

Manuscript ID number: PONE-D-20-19334R1

We would like to thank the reviewers for their valuable time and useful contributions to the manuscript. We have carefully considered and addressed each of the comments provided and have made revisions in line with their recommendations. Herein, we are presenting a more comprehensive and improved manuscript for publication.

Point-by-point responses to each of the reviewers’ comments are included within the revised submission. We thank you for considering our revised manuscript for publication and look forward to your reply.

Yours sincerely,

Samuel Schumacher

Deputy Head of TB

FIND

[samuel.schumacher@finddx.org](mailto:samuel.schumacher@finddx.org)

T +41 (0) 22 710 05 91

M +41 (0) 79 911 45 91

| Reviewer | Point | Line | Reviewer Comment | Response |
| --- | --- | --- | --- | --- |
| 1 | 1 | N/A | 3 of the 4 major points I raised were dismissed without substantive discussion. I have asked to have the manuscript reviewed by a statistician | We apologise to the reviewer that they felt their points were dismissed without a proper discussion into issues raised. We too have had the manuscript reviewed by a statistician and our reply is as follows:  We think the methodology the reviewer proposes broadly makes sense. We agree that in large and complex analyses data-driven variable selection and correction for selection bias are important facets of sound methodology with studies such as ours.  However, these techniques are often aimed at improving the validity of analysis concerning associations with secondary outcomes that go beyond the main outcome of disease. Furthermore, as we have previously mentioned, they are typically used to address circumstances in which cases and controls are selected with unequal probability, a situation that does not strictly apply to our study. We are also of the opinion our regression analysis is a relatively “simple” one, largely designed to be illustrative, and as such we don’t see significant additional value in overcomplicating it with the suggested methodology, especially as the number of variables included *a priori* was small. While our ROC curve analysis is clearly more central to the study’s results, we feel the explanation above justifies the methodology we have used. |
| 2 | 1 | Table 1 | In Table 1 / Smear status, we are told there were 289 participants S+C+ and 102 S-C+, whereas in Table 3 to the left of S+C+ we are given n=663 and for S-C+ we are given n=476; these numbers are not the numbers for S+C+ and S-C+ and must refer to [S+C+ plus all TB test neg] and [S-C+ plus all TB neg] but this is not immediately clear on first reading (or second reading I confess) - it might help to explicitly state in the line just below the table "Smear-positive and smear-negative participants were each assessed against all TBneg (n= 374)", or to insert another line in the table for All TB test neg | We agree with the reviewer that this is not clear and have amended the legend below the table to include how these numbers were arrived at, in line with the reviewer’s recommendations. |
| 2 | 2 | 306-309 | In the Discussion you note (in lines 306-309) that using a CRP cut off of 8 mg/L would result in approximately 26% of culture positive cases being missed. I expect this 26% to accord with the numbers given in Supplementary Figure 1 which relates to the use of the 8 mg/L cut off. In S Fig 1 it appears that a total of 79 culture + cases occurred among those with CRP <8 mg/L (with the total of all culture positive cases being 79 + 312 = 391) which looks like 20.2% (79/391) were missed (because CRP <8). Where does 26% come from? Is there a typographical error and you mean to continue referring to an 18 mg/L cut off, not an 8 mg/L cut off as the text currently states? (In that case it would obviously have nothing to do with S Fig 1.) | We agree with the reviewer that these numbers do not concord with each other.  The reason for the use of 26% was because this statement in the discussion related to the use of the Xpert reference standard not the microbiological reference standard – 26% was calculated on the basis of the number of participants that had been tested with Xpert MTB/Rif who would have been missed diagnoses according to the use of the algorithm specified in the “CRP Triage Algorithm” section of the results. We have amended the discussion to make this clearer.  However, the value of 26% itself was incorrect – as pointed out by a separate reviewer, we mistakenly included indeterminate Xpert results in the denominator to arrive at this value. For all other analysis in this paper, we had excluded indeterminate Xpert results (our intention). We have clarified this intention in the methods and amended all relevant areas in the results and discussion to correct this error. We apologise for the confusion and hope these amendments address the reviewer’s comment. |
| 4 | 1 | Table S6 + CRP Triage Algorithm | The number requiring confirmatory Xpert test was 59.4% - this is not “almost half” as reported because this is almost 60%. According to page 17, 60/226 TBpos participants with Xpert results (26.5%) would be missed. However, in Table 1, there were 183 Xpert+ and 38 Xpert- = 221 Xpert results among TBpos participants. Furthermore, it was stated that “if Xpert MTB/Rif were implemented for all without CRP triage, 38 (16.8%) participants would be missed”. This is correct if indeed the number of Xpert results is 226 but not 221 (38/221 = 17.2%). However, the sensitivity reported in Table S6 is 82.8% which means 17.2% will be missed. Please check these numbers or am I missing something? If I’m correct please also fix references to these numbers in the discussion | Regarding the reviewer’s first point, the authors intention was to convey that almost half of participants did not require a confirmatory test. However, given that this may be deemed inaccurate, we have changed the manuscript to read that 40% (100-59.4% = 40.6% ) did not require confirmatory testing. We hope this addresses the reviewer’s first point.  Regarding the second half of the reviewer’s comment, the confusion arises due to indeterminate Xpert MTB/Rif test results. In 5 cases where the Xpert result was indeterminate, the culture result was positive, hence the differing denominators of 221 in the table but 226 on page 17. The use of 226 as the denominator was a mistake as we decided to exclude indeterminate Xpert results from the analyses we completed on Xpert performance. As a result, we have changed the relevant parts of the manuscript to reflect the numbers and percentages that are incorrect. We have also added a sentence to the Methods section to clarify how indeterminate Xpert MTB/Rif results were handled. |
| 4 | 2 | Abstract | please use clinical or diagnostic accuracy as the term “clinical utility” is often used to refer to impact on patient outcomes | The authors agree with the reviewer and have made the suggested change |
| 4 | 3 | Abstract | were the 765 serum samples from 765 adults presenting with respiratory symptoms or were there multiple samples from some patients? The results in the main text reported 765 participants but not clear in the abstract | Each sample was from a single study participant. The authors have amended the wording in the abstract to reflect this. |
| 4 | 4 | Abstract | is MGIT a well know acronym? | MGIT is the most commonly used mycobacterial liquid culture medium, but the authors recognise that best practice is define all acronyms at first usage and so have modified the abstract to replace “MGIT” with “liquid” in line with the reviewer’s comment. |
| 4 | 5 | Abstract + elsewhere | “pooled sensitivity” The use of ‘pooled’ is confusing and misleading. The results are from a single study and not from a meta-analysis or from combining results from multiple cohorts in one study. A more appropriate word is ”overall” but only needed when there is ambiguity about whether you are referring to a subgroup or all patients | We agree with the reviewer and have changed all relevant sections of the manuscript |
| 4 | 6 | Abstract | Please change “operator” to “operating” in “Receiver Operator Characteristic” | We agree with the reviewer and have changed all relevant sections of the manuscript |
| 4 | 7 | Abstract discussion and main discussion | I can’t see the promise since it failed to meet the minimum TPP value of 90% sensitivity even in a retrospective study and results were not reported in the abstract for HIV+ individuals. Even for PLHIV, is it really promising given the poor specificity that is no better than tossing a coin when assessed against culture? | We agree with the reviewer that overall performance of the test was suboptimal with respect to the MRS and the target TPP. However, when different CRP cutoff-points were explored, performance of the test in HIV positive participants did near TPP; at a cutoff-point of 23mg/L sensitivity was 85.6% and specificity was 69.6% for HIV+ participants as we stated in the subgroup section of the results. Further to this, were an Xpert reference standard to be used as would be more likely in many low-income settings, performance of CRP relative the TPP improves albeit due to a reduction in sensitivity of the confirmation test. Our supplementary data (Table S3) shows that at a cutoff-point of 18mg/L using the XRS, overall sensitivity and specificity were 84.7% and 69.5% respectively. Using difference cutoff-points for HIV+ and HIV- participants produced similar performances in both subgroups. We would argue that these results are sufficiently close enough to the TPP to warrant further investigation into the role of CRP as a triage test in this cohort of patients and to justify our assertion that they show promise. |
| 4 | 8 | Study population | the study is described as a nested case control study. Is this correct since not all the cases were included unless I’m missing something? | The authors feel that the present study does qualify as a nested case-control design. While it is very true that nested case-control designs often include all cases from the parent cohort as the reviewer states, this is not necessary to define a case-control design. The key defining characteristic of a nested case-control study is that a random sample of cases and controls are selected from a fully enumerated cohort, which is the case here. This is supported in Rothman KJ, Greenland S, Lash TL, editors. Modern epidemiology. Lippincott Williams & Wilkins; 2008. |
| 4 | 9 | Sample size calculation | “targeted performances of the index test” please specify what they are for sensitivity and specificity – were these the values in the TPP? Were the same estimates used for the HIV+ subpopulation? | We agree and have made the changes suggested in the reviewer’s comment |
| 4 | 10 | Statistics | Suggest rephrasing the sentence “Results were reported as a point estimate with 95%CI calculated using Wilson’s method for pre-defined CRP cutoff-points” as it sort of implies Wilson’s method is for the cutoffs rather than CIs. Selecting variables based on statistical significance in univariable analysis is discouraged while the use of backward elimination is encouraged. Given the exploratory nature of the analysis and because it was pre-specified, I will not make this a major issue but please note for future analyses. How did you deal with missing data or was your analysis a complete case analysis? Was the same dataset used for the univariable and multivariable analysis as the dataset will be different depending on whether or not each variable had some missing data. | We agree with the reviewer on the first point regarding the structure of the quoted sentence and have amended the manuscript to make this clearer.  We note the reviewer’s comments surrounding the univariable analysis with thanks and we will incorporate this advice into future analyses.  Our analysis was a complete case analysis, with the same dataset used for both univariable and multivariable analyses. We have amended the methods section to clarify this. |
| 4 | 11 | Result | please give the number of women so it reads “…X (40.1%) were women…”. Also give % for “…527/765…” so it reads “…527/765 (X%)…” There is a typo in “Night sweats” should be “night sweats” | We agree with the reviewer and have amended these points |
| 4 | 12 | Page 14 | Please give CIs for all estimates of sensitivity and specificity reported on page 14. Please write sens and spec in full on this page when referring to the TPP. | We agree with the reviewer and have amended the manuscript with the suggested changes. |
| 4 | 13 | S1_Fig | Figure S1_Fig did not download properly so I cannot comment on it | We will attempt to re-upload this file to see if it assists the reviewer in downloading it. |
| 4 | 14 | Page 15 | Please cross-reference Table S2 for the results against XRS that were reported on this page | We agree with the reviewer and have amended this |
| 4 | 15 | Results – subgroup analysis | results of HIV+ and HIV- patients were compared but specificity not stated for HIV positives especially as the sensitivity of >90% was highlighted. Please give the CIs along with the estimates reported in this section. | We agree with the reviewer and have amended this section of the results to include 95%CI and the requested specificities from Table 3. |
| 4 | 16 | Throughout | Inconsistent use of the terms HIV+/HIV-, HIV-positive/HIV-negative – please choose one pair and use consistently throughout. Are you using HIV+ or PLHIV? | We agree with the reviewer that our use of HIV+ vs positive etc is inconsistent and so we have amended the manuscript to use the nomenclature HIV+ and HIV-. |
| 4 | 17 | Page 17 | “…difference in the AUC between these two populations was not significant.” Did you formally test this? I interpret this to be the case with the use of term “not significant”. | We did formally test this but did not report the p value for brevity’s sake. |
| 4 | 18 | Discussion | “…but fell 13% short of the specificity of the TPP…” I think you mean 13 percentage points rather than 13% because you are referring to an absolute difference rather than 13% of the TPP specificity value. Same too later in that paragraph where you mentioned “10% more” | We did mean percentage points and not percent and thank the reviewer for pointing this out. We have changed the relevant parts of the manuscript. |
| 4 | 19 | Discussion | earlier stated that the analysis by study site was pre-specified (in methods as country of origin of the sample,) but in the discussion stated as post-hoc. Which is it? | Analysis by country of origin was post-hoc and the methods section was incorrect. We have amended the methods to reflect this. |
| 4 | 20 | Results/Table | I couldn’t figure out why the numbers do not add up to the total for smear positive culture positive and smear negative culture positive. | We have amended the legend to the relevant table to make it clearer as to how the figures were reached. |
| 4 | 21 | Table 3 and supplementary material | For complete reporting, please give the number of cases in addition to n (e.g. n/N) in all main and supplementary tables like Table 3 that report sensitivities and specificities. This will enable readers to easily derive 2x2 tables if they wish to do so. | We agree and have amended Table 3 and the supplementary tables S2 and S4 in line with the reviewer’s suggestion. |
| 4 | 22 | Supplementary material | The STARD checklist was used to report the study but was not included as a supplementary file. Not required by the journal? | The reviewer is correct that this was not required by the journal. However, we are happy to upload the guidelines as a supplementary file. |
