# Supplementary material for: Evaluation of the diagnostic performance of laboratory-based c-reactive protein as a triage test for active pulmonary tuberculosis
Source: PLoS One. 2021 Jul 12;16(7):e0254002. doi: 10.1371/journal.pone.0254002 (PMC8274836; doi:10.1371/journal.pone.0254002)
Supplement: Attachment — Submitted filename: CRP for TB Triage Reviewer comments with replies v3.0.docx [file pone.0254002.s017.docx]

Foundation for Innovative Novel Diagnostics Headquarters

Campus Biotech

9 Chemin des Mines

1202 Geneva

Switzerland

08.05.21

Dear Editor of PLOS One,

It is with pleasure that we are submitting our revised article to PLOS One entitled:

**Evaluation of the diagnostic performance of laboratory-based C-reactive protein as a triage test for active pulmonary tuberculosis**

Manuscript ID number: PONE-D-20-19334R1

We would like to thank the reviewers again for their valuable time and useful contributions to the manuscript. We have addressed each of the remaining comments provided and have made the suggested revisions. We now present a revised manuscript that we hope both you and the reviewers find suitable for publication

Point-by-point responses to each of the reviewers’ comments are included within the revised submission. We thank you for considering our revised manuscript for publication and look forward to your reply.

Yours sincerely,

Samuel Schumacher

Deputy Head of TB

FIND

[samuel.schumacher@finddx.org](mailto:samuel.schumacher@finddx.org)

T +41 (0) 22 710 05 91

M +41 (0) 79 911 45 91

**Reviewer 2:**

**Comment:**  Line 28: Since the word data is the plural of datum, the sentence should read "...limited data are available..."

**Response:** We agree with the reviewer and have corrected this grammar in the abstract

**Comment:** Line 317: the word 'quarter' is misspelled (appears as quater).

**Response:** We thank the reviewer for bring our attention to this! We have changed the spelling.

**Comment:** Line 372-3: Since 'data' is plural, the sentence should read "Previous data assessing the role of CRP in the diagnosis of TB have largely been...", not 'has largely been'.

**Response:** We agree with the reviewer and have corrected this grammar.

**Reviewer 4:**

**Comment:** Where you have replaced "a pooled" with "a overall", please correct to "an overall"

**Response:** Many thanks to the reviewer for bringing this to our attention. We have made this correction to the relevant points in the manuscript have corrected a similar grammatical mistake we identified elsewhere.

**Comment:** Discussion: in the results you gave 40.6% but stated 40% in the discussion. Please write "about 40%" of give exact value of 40.6%.

**Response:** We agree with the reviewer and have altered the relevant bit of the discussion to read “about 40%”.

**Comment:** Discussion: "quater" should be "quarter".

**Response:** Similar to point 1, thanks again to the reviewer for bringing this to our attention. Overall, a lesson in making sure one does a final spell check!
